# Plant vigour QTLs co-map with an earlier reported QTL hotspot for drought tolerance while water saving QTLs map in other regions of the chickpea genome

**DOI:** 10.1186/s12870-018-1245-1

**Published:** 2018-02-06

**Authors:** Kaliamoorthy Sivasakthi, Mahendar Thudi, Murugesan Tharanya, Sandip M. Kale, Jana Kholová, Mahamat Hissene Halime, Deepa Jaganathan, Rekha Baddam, Thiyagarajan Thirunalasundari, Pooran M. Gaur, Rajeev K. Varshney, Vincent Vadez

**Affiliations:** 1 International Crops Research Institute for the Semi-Arid Tropics (ICRISAT), Greater Hyderabad, Telangana, India; 2 Bharathidasan University, Tiruchirappalli, Tamil Nadu India; 3 Institut de Recherche pour le Developpement (IRD), Université de Montpellier – UMR DIADE, 911 Avenue Agropolis, BP 64501, 34394 Montpellier cedex 5, France

**Keywords:** Phenotyping, Plant vigour, Transpiration rate, Quantitative trait loci (QTL), “*QTL-hotspot*”, Drought stress

## Abstract

**Background:**

Terminal drought stress leads to substantial annual yield losses in chickpea (*Cicer arietinum* L.). Adaptation to water limitation is a matter of matching water supply to water demand by the crop. Therefore, harnessing the genetics of traits contributing to plant water use, i.e. transpiration rate and canopy development dynamics, is important to design crop ideotypes suited to a varying range of water limited environments. With an aim of identifying genomic regions for plant vigour (growth and canopy size) and canopy conductance traits, 232 recombinant inbred lines derived from a cross between ICC 4958 and ICC 1882, were phenotyped at vegetative stage under well-watered conditions using a high throughput phenotyping platform (LeasyScan).

**Results:**

Twenty one major quantitative trait loci (M-QTLs) were identified for plant vigour and canopy conductance traits using an ultra-high density bin map. Plant vigour traits had 13 M-QTLs on CaLG04, with favourable alleles from high vigour parent ICC 4958. Most of them co-mapped with a previously fine mapped major drought tolerance “*QTL-hotspot*” region on CaLG04. One M-QTL was found for canopy conductance on CaLG03 with the ultra-high density bin map. Comparative analysis of the QTLs found across different density genetic maps revealed that QTL size reduced considerably and % of phenotypic variation increased as marker density increased.

**Conclusion:**

Earlier reported drought tolerance hotspot is a vigour locus. The fact that canopy conductance traits, i.e. the other important determinant of plant water use, mapped on CaLG03 provides an opportunity to manipulate these loci to tailor recombinants having low/high transpiration rate and plant vigour, fitted to specific drought stress scenarios in chickpea.

**Electronic supplementary material:**

The online version of this article (10.1186/s12870-018-1245-1) contains supplementary material, which is available to authorized users.

## Background

Chickpea (*Cicer arietinum* L.), the second most important grain legume crops in the world [1], is widely cultivated on residual soil moisture in the arid and semi-arid agricultural systems of the world. Terminal water deficit is one of the major constraints limiting the chickpea crop productivity [2] and has been reported to cause yield losses upto 50% in chickpea [3].

Deeper and more profuse rooting has been hypothesized to be the major factor contributing to yield increase under water limited environments in chickpea, where the assumption was made that these root traits would increase water extraction [4–8]. However it was also shown that chickpea genotypes with deeper and more profuse rooting did not extract more water from the soil profile [9]. Rather, tolerant chickpea genotypes turned out to be those able to somewhat limit water use at vegetative stage and making more water available for the grain filling period [9, 10]. Similar results have been reported in other crops (e.g. in pearl millet [11], in sorghum [12]). Therefore, the central hypothesis of the present study is that, given the limited seasonal water budget, any trait allowing water conservation during vegetative growth (e.g. canopy conductivity, canopy size and development) extends the duration of water extraction during pod filling and so increases productivity of chickpea crop under terminal water stress [9–11, 13–15].

In chickpea, the availability of large scale genomic resources has paved the way to dissect the mechanisms underlying various stresses adaptations [16, 17]. A recent mapping study in chickpea reported a genomic region on CaLG04 referred as a “*QTL hotspot*” that harbours several drought tolerance traits including rooting depth [18]. Introgression of this region into elite variety JG 11 improved yield under drought [19]. This reported “*QTL hotspot*” region (spanning ~ 29 cM) was originally associated with seven SSR markers [20]. Further, this “*QTL-hotspot*” region was refined to~ 14 cM, with additional 49 SNP markers, [20] using genotyping-by-sequencing (GBS). Skim sequencing (with bin used as markers, based on recombination break points) approach then allowed to fine map this region to ~ 300 Kb [21]. An intriguing feature of the preliminary steps of this research was also the mapping of a major QTL for shoot weight on CaLG04, which co-mapped with a QTLs for root traits (depth and length density), from a study where these traits were assessed in PVC tubes [22]. Interestingly, the percentage of phenotypic variation explained by this QTL was more for the shoot dry weight than for the root traits, suggesting that this QTL region was a QTL for vigour, but this hypothesis was not followed further.

In chickpea, the studies of physiological traits allowing water conservation (e.g. canopy conductivity, canopy size & development; [9, 10]) are very scarce, partially because an accurate assessment of leaf area is a rate limiting step. Recognizing this obstacle, a high throughput phenotyping platform was developed to measure canopy development traits [23]. The high throughput platform was used to phenotype the RIL population (ICC 4958 × ICC 1882), from which the “*QTL-hotspot*” was reported, for plant vigour traits (leaf area, plant height, rate of leaf area increase) and water saving traits (conductance), as a mean to re-investigate the map location of these traits with regards to the QTL hotspot earlier identified [18].

Therefore, the overall objective of this study was: i) to assess the phenotypic variation in traits involved in the control of plant water use either from canopy development or canopy conductance, and explore their functional associations in a RIL mapping population previously used for mapping the “drought tolerance QTL” (ICC 4958 × ICC 1882), ii) to map these drought adaptive traits and assess their interactions, iii) to conduct comparative mapping study using differently saturated genetic maps.

## Results

Based on trait functionality, these were clustered (Clustering analysis) and grouped into two major clusters: (i) a cluster of plant vigour traits [Plant vigour score (VIG), 3D-leaf area (3D-L), projected leaf area (PL), plant height (PH), 3D-leaf area growth rate (3D-LG), projected leaf area growth rate (PLG), plant height growth rate (PHG), shoot dry weight (SDW), leaf area index (LAI), specific leaf weight (SLW) and specific leaf area (SLA)]; and (ii) a cluster of traits related to canopy conductance [Transpiration (T), evapotranspiration (eT), transpiration rate (TR), evapotranspiration rate (eTR) and the residuals between 3D and projected leaf area (R-3D/PLA, a trait that was interpreted to represent the canopy structure)] (Table 1; Additional file 1).

**Table 1:** Summary on traits phenotyped using high throughput plant phenotyping platform (LeasyScan). Summary include trait name, trait code, trait type, year of phenotyping, replication, and measurement methods

No. of traits	Trait name	Trait code	Trait type	Year of phenotyping	Replication	Measurement method
1	Plant vigour	VIG	Plant vigour	Nov-Dec-2015	4	Visual eye scoring
2	Projected Leaf area (cm^2^)	PL	Plant vigour	Nov-Dec-2014 & 2015	3 & 4	LeasyScan-Plant eye camera
3	Projected Leaf area growth rate (cm^2^ day^− 1^)	PLG	Plant vigour	Nov-Dec-2014 & 2015	3 & 4	LeasyScan data derived
4	3-Dimentional (3D) Leaf area (mm^2^)	3DL	Plant vigour	Nov-Dec-2014 & 2015	3 & 4	LeasyScan-Plant eye camera
5	3-Dimentional (3D) Leaf area growth rate (mm^2^)	3DLG	Plant vigour	Nov-Dec-2014 & 2015	3 & 4	LeasyScan data derived
6	Leaf area index	LAI	Plant vigour	Nov-Dec-2014 & 2015	3 & 4	LeasyScan data derived
7	Shoot dry weight (g)	SDW	Plant vigour	Nov-Dec-2014 & 2015	3 & 4	LeasyScan & gravimetric data derived
8	Specific leaf area (g mm^2^)	SLA	Plant vigour	Nov-Dec-2014 & 2015	3 & 4	LeasyScan & gravimetric data derived
9	Specific leaf weight (mg mm^2^)	SLW	Plant vigour	Nov-Dec-2014 & 2015	3 & 4	LeasyScan & gravimetric data derived
10	Residuals from 3-D & projected Leaf area (cm^2^)	R-3D/PLA	Canopy structure	Nov-Dec-2014 & 2015	3 & 4	LeasyScan data derived
11	Plant height (cm)	PH	Plant vigour	Nov-Dec-2014 & 2015	3 & 4	LeasyScan-Plant eye camera
12	Plant height growth rate (cm day^−1^)	PHG	Plant vigour	Nov-Dec-2014 & 2015	3 & 4	LeasyScan data derived
13	Evapotranspiration (g)	eT	Canopy conductance	Nov-Dec-2014 & 2015	3 & 4	Gravimetric pot weighing
14	Evapotranspiration rate (mg mm ^2^ day^−1^)	eTR	Canopy conductance	Nov-Dec-2014 & 2015	3 & 4	Gravimetric & LeasyScan-data derived
15	Transpiration (g)	T	Canopy conductance	Nov-Dec-2014 & 2015	3 & 4	Gravimetric data derived
16	Transpiration rate (g cm ^2^ day^−1^)	TR	Canopy conductance	Nov-Dec-2014 & 2015	3 & 4	Gravimetric & LeasyScan-data derived

### Phenotypic analysis

#### Plant vigour related traits

##### Summary statistics

**Fig. 1:**
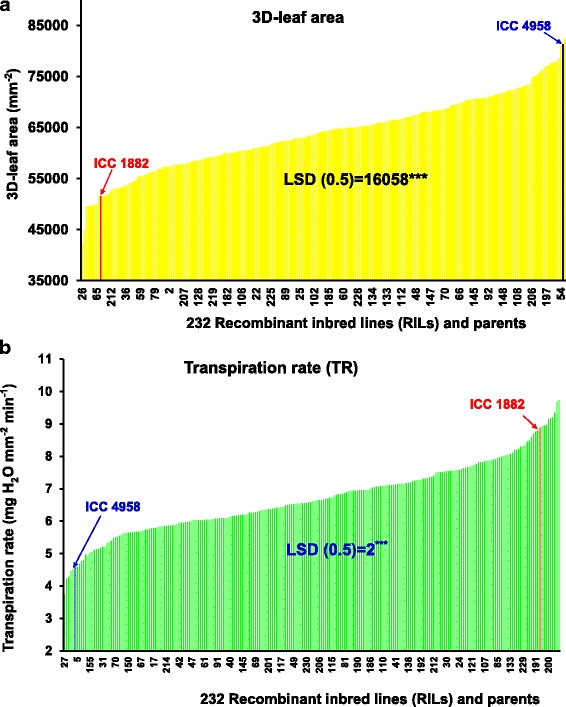
Range of variation for plant vigour and canopy conductance related traits from LeasyScan. Range of variation in **a**) 3D-Leaf area (mm^− 2^) and **b**) transpiration rate (TR; mg H_2_O mm^− 2^ min^− 1^) in 232 RILs and parents (ICC 4958 & ICC 1882) at 28 DAS under well watered conditions

The two parental genotypes (ICC 4958 and ICC 1882), as well as RILs, showed significant differences in plant vigour traits in both years (Table 2). For example, 3D-Leaf area (3D-L) was among those showing the largest variation, i.e. a 5-fold range variation in both years (Fig. 1a & Table 2). Continuous variation and normal frequency distribution were found for plant vigour traits (Additional file 2 A, B, C &D). Additional file 3 A & B showed 3D-leaf area & plant height development dynamics of parental lines. The high vigour parent ICC 4958 had faster leaf area and plant height development (canopy development) than low vigour parent ICC 1882.

**Table 2:** ANOVA results for the 16 traits phenotyped using high throughput plant phenotyping platform (LeasyScan). F represents probability; SE represents the standard error; LSD represents least significant difference and h^2^ represents the heritability values

			Parents				Progenies					
Trait No.	Traits code	Year	ICC 4958	ICC 1882	Significance	LSD	Variation in RILs	Grand mean	Significance	S.E	LSD	h^2^ (%)
1	VIG	2015	5	2.0	0.01	1.0	2.0 - 5.00	3.718	<.001	0.50	0.97	73
2	3DL	2014	46,497	25,389	0.01	16,147	14,237 -71,290	35,549.7	<.001	5575.00	10,956	76
2	3DL	2015	54,684	33,353	0.01	19,884	14,292 - 68,103	40,299	<.001	6285.00	12,339.5	89
3	3DG	2014	3079	2031	0.05	674	1207 - 4461	2397	<.001	311.80	612.7	72
3	3DG	2015	2298	1774	0.05	407	310.5 - 4487	2146	<.001	328.00	643	85
4	PL	2014	435	252	0.01	127	175 - 561	323	<.001	34.50	68.4	50
4	PL	2015	515	354	0.01	174	260 - 649.3	447	<.001	43.50	85.3	70
5	PLG	2014	68	38	0.01	27	−0.079 - 6.7	2.191	<.001	0.73	1.42	37
5	PLG	2015	19	13	0.01	7.4	4.14 - 43.15	18.43	<.001	4.80	9.4	41
6	PH	2014	110	76	0.01	29	54 - 150	96.87	<.001	4.41	8.66	96
6	PH	2015	126	72	0.01	26	47.41 - 198.3	102.7	<.001	9.20	18.1	88
7	PHG	2014	3.1	1.47	0.05	1.2	12.15 - 99.45	57.96	<.001	11.40	22.4	62
7	PHG	2015	2.1	0.97	0.01	1.1	−6.12 - 4.30	1.45	<.001	0.58	1.14	57
8	LAI	2014	0.60	0.42	0.05	0.1	0.18 - 0.79	0.383	<.001	0.0566	0.1113	59
8	LAI	2015	1.21	0.79	0.01	0.4	0.5882 - 1.399	0.988	<.001	0.11	0.21	45
9	R-3D/PLA	2014	0.19	−8.18	ns	22	−95.54	−0.401	<.001	8.16	16.04	68
9	R-3D/PLA	2015	44	68	0.05	17	−26.4 - 160.6	62.54	<.001	16.55	32.53	51
10	SDW	2014	20	12.9	0.01	6.2	8.66 - 28.97	15.78	<.001	1.21	2.38	86
10	SDW	2015	18	11.3	0.01	4.5	6.523 - 25.09	14.24	<.001	2.60	5.12	60
11	SLA	2014	4651	3975	0.05	628	1403 - 9774	4221	<.001	951.80	1870.4	66
11	SLA	2015	3287	2559	0.01	369	543.7 - 7116	2471	<.001	622.00	1222.3	64
12	SLW	2014	0.21	0.25	0.05	0.04	0.102 - 0.7126	0.2616	<.001	0.06	0.12	70
12	SLW	2015	0.73	0.33	0.01	0.25	0.1525 - 1.549	0.4849	<.001	1.22	0.24	72
13	Et	2014	37	24	0.05	4.89	13.92 - 37	22.01	<.001	2.65	5.208	65
13	eT	2015	74.46	58.46	0.01	10.44	39.83 - 108	73.21	<.001	7.9	15.6	53
14	eTR	2014	0.537	1.156	0.01	0.267	0.306 - 1.532	0.771	<.001	0.119	0.233	25
14	eTR	2015	1.278	3.00	0.01	0.539	0.918 - 3.473	1.611	<.001	0.264	0.519	25
15	T	2014	20.33	13.00	0.01	3.069	5.074 - 34.42	16.84	<.001	3.11	6.12	62
15	T	2015	50.26	34	0.01	6.75	17.13 - 88.78	52.28	<.001	6.63	13.02	70
16	TR	2014	0.00047	0.00090	0.01	0.00031	0.000289 - 0.00089	0.00058	0.004	0.000083	0.000163	41
16	TR	2015	0.00046	0.00086	0.01	0.00021	0.00034 - 0.00189	0.00111	<.001	0.000167	0.000328	57

According to Rabinson et al. [24], heritability (*h*^*2*^%) is classified as low (0-30%), moderate (30-60%) and high (> 60%). Most of the plant vigour related traits had *h*^*2*^% in the range of 60- 90% (e.g. PH, 3DL, 3D-LG, SLW and SDW; Table 2). Among these, plant height (PH) and 3DL showed highest heritability [PH (87.5 and 88%) and 3DL (76 and 89%) in 2014 and 2015 respectively].

#### Canopy conductance traits

##### Summary statistics

**Fig. 2:**
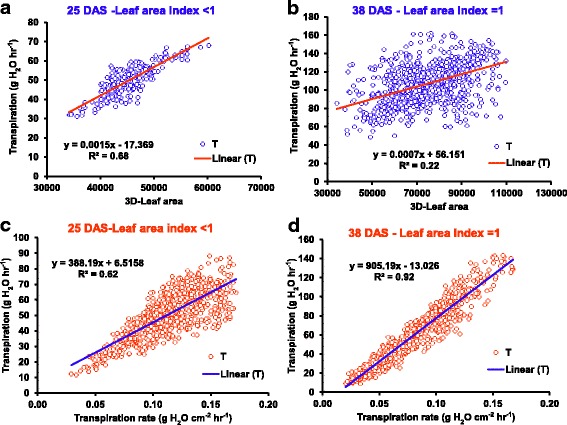
Relationship between plant vigour (3D-L) and canopy conductance related traits (T &TR) from LeasyScan. **a** represents the relationship between transpiration and 3D-leaf area at 25 DAS (Leaf area index > 1). **b** represents the relationship between transpiration and 3D-leaf area at 38 DAS (Leaf area index = 1). **c** represents the relationship between transpiration and transpiration rate at 25 DAS (Leaf area index > 1). The (**d**) represents the relationship between transpiration and transpiration rate at 38 DAS (Leaf area index = 1)

The two parental genotypes (ICC 4958 and ICC 1882) and RILs (progenies) showed significant difference for all canopy conductance traits (T, TR, eT, eTR & R-3D/PLA) in both years (2014-2015; Table 2). For example, T was one among those showing the largest phenotypic variation, i.e. a 5-fold range variation in both years (Table 2). In addition, TR also showed 2-fold range of variation (Fig. 1b). Continuous variation and normal frequency distribution was found for all traits (Additional file 2 E, F, G & H - data not shown for R-3D/PLA). Transpiration and 3D-leaf area were tightly correlated (r^2^ = 0.68) until the LAI reached a value of 1 (25 DAS). Thereafter, this relationship became much weaker (r^2^ = 0.22) when the plants reached an LAI between 1 and 2 (38 DAS; see Fig. 2 a & b). At this stage, TR became much more closely related to T (r^2^ = 0.92), whereas this relationship was weaker (r^2^ = 0.62) when the LAI was less than 1 (25 DAS; see Fig. 2 c & d). Hence it was interpreted that at a low LAI, leaf area was the main driver of T. By contrast, at a high LAI, TR was the main driver of T. Since the average VPD during the transpiration measurement was high (3.76 kPa), this was interpreted to be caused by TR differences under high VPD.

Most of the canopy conductance traits had low to medium (25 to 68%) heritability (e.g. TR, eT, eTR and R-3D/PLA), except T (high *h*^*2*^ range; 62 and 70% in 2014 and 2015 respectively) (Table 2).

#### Trait correlation and their relationships

##### Simple Pearson correlation analysis

Phenotypic correlation coefficients of ICC 4958 x ICC 1882 population are presented in Additional file 4. As expected there were strong relationships within both groups of traits, but also between traits across groups. As expected, 3DL, LAI and SDW (plant vigour traits) were positively correlated with T and eT (canopy conductance traits), whereas 3DL, PL, LAI and SLA were negatively correlated with TR (see Additional file 4). Interestingly, most plant vigour traits were negatively correlated with R-3D/PLA (Canopy structure). By contrast, R-3D/PLA was positively correlated with TR and eTR. A significant correlation was observed among plant vigour traits. For example, plant vigour score (VIG) was significantly correlated with PH (0.73, *P* = 0.0001), PHG (0.62, P = 0.0001), 3DL (0.46, P = 0.0001), LAI (0.47, P = 0.0001), SDW (0.44, P = 0.0001) (Additional file 4). In addition, a significant correlation was observed among canopy conductance traits. For example, TR was well correlated with eT (0.63, P = 0.0001), eTR (0.61, P = 0.0001) and T (0.44, P = 0.0001) (Additional file 4).

##### Principal component analysis (PCA)

A principal component analysis was used to identify the relationships between parameters, and group these in a more comprehensive manner. Three principal components (PC) explained 62% of the total variation observed among the RIL population, using BLUPs phenotypic data across years (Additional files 5 and 6). PC1 (34%) had a strong positive loading from SLA and a strong negative loading from 3DL (Additional file 6), which agrees well with the strong negative correlations between these traits (Additional file 4). PC2 (17%) had a strong positive loading from PL and 3DL (plant vigour traits), whereas most canopy conductance traits had a strong negative loading. This also agreed well with the strong negative correlations between plant vigour (PL and 3DL) and canopy conductance (TR and eTR) traits (Additional file 4). PC3 (13%) had a strong positive loading from VIG, PH, PHG, SLA and T, whereas growth rate traits (3D-LG. PLG), canopy structure (R-3D/PLA) and SLW had strong negative loading (Additional file 6). This agreed well with the strong negative correlations between most of the plant vigour and canopy structure (R-3D/PLA) traits (Additional file 4).

### Genomic analysis

#### Plant vigour traits

##### QTL analysis for single locus

Plant vigour related traits mapped predominantly on CaLG04. One M-QTL [LOD 36.7 & PVE 53%] for VIG was identified in CaLG04 within the reported refined “*QTL-hotspot*” region (~ 300 Kb; Additional file 7). For 3DL, three M-QTLs were identified, all (LOD 6-10 & PVE 11-18%) being found in both years within the earlier reported refined “*QTL-hotspot*” region with the favourable allele from ICC 4958 (Additional file 7). For PL, three QTLs were identified within the “*QTL-hotspot”*, with the favourable allele from ICC 4958 (Additional file 7). Among these, one was a M-QTL (LOD 6 & PVE 11%) and the remaining two were minor QTLs with PVE 8-9% (Additional file 7). For LAI, one M-QTL (LOD 6 & PVE 11%) was identified within the “*QTL-hotspot”* with the favourable allele from ICC 4958 (Additional file 7). Two minor QTLs were identified in CaLG04 (2 QTLs) (Additional file 7). For SDW, one M-QTL (LOD 9 & PVE 18%) was found with favourable allele from ICC 4958 within the “*QTL-hotspot”* (Additional file 7), and one minor QTL was identified in CaLG04 (Additional file 7). For PH, three M-QTLs (LOD 20-22 & PVE 34-39%) were identified in CaLG04 within the “*QTL-hotspot”* with favourable allele from ICC 4958, and one minor QTL was identified in CaLG04 (Additional file 7). For PHG, three M-QTLs (LOD 7-14 & PVE 13-23%) were found in CaLG04 within the “*QTL-hotspot”* with favourable allele from ICC 4958, and one minor QTL was identified in CaLG04 (Additional file 7). For SLA, three minor QTL were found in CaLG04 (PVE 4-8%; Additional file 7). Few major and minor QTLs from other CaLGs of plant vigour related traits were presented in Additional file 7.

##### Interaction QTL analysis for multiple loci

Plant vigour and canopy conductance related traits, epistatic QTL (E-QTLs) interactions were analyzed using genotype matrix mapping (GMM). In this section, only selected strongest epistatic QTL (E-QTLs) interactions and high F values with RILs number higher than 10 are discussed (Additional file 8). Additional E-QTLs interactions (Lower F values and RIL number and PVE %) for plant vigour and canopy conductance related traits were found and are shown in Additional file 9. Many E-QTLs interactions were identified for plant vigour traits (VIG, 3D-L, PL, PH, 3D-LG, PLG, PHG, SDW, LAI, SLW and SLA) and these are listed in Additional file 8.

**Fig. 3:**
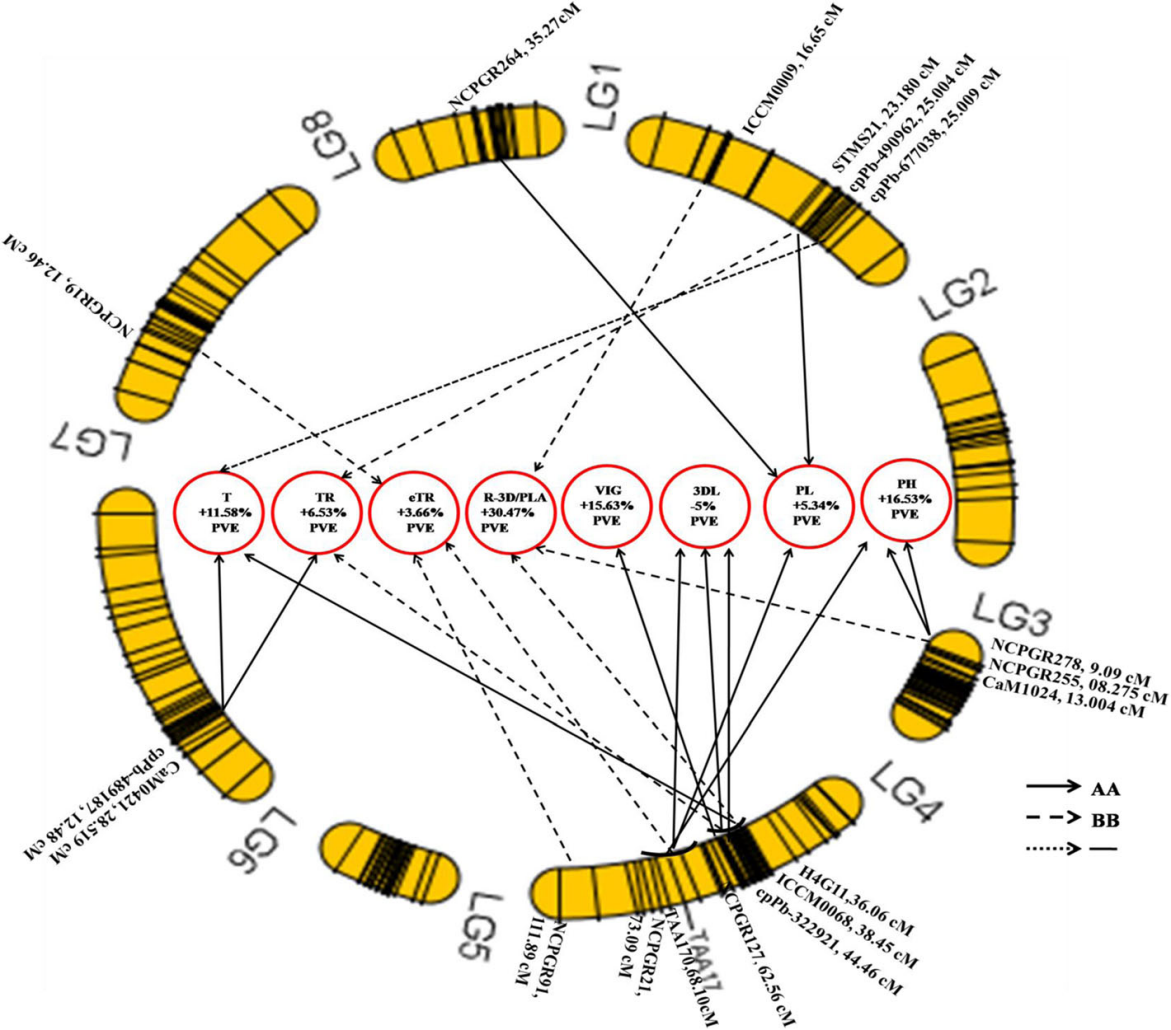
QTL interactions of plant vigour and canopy conductance related traits using genotype matrix mapping analysis. Solid lines represent the positive allele from high vigour parent ICC 4958 and dashed lines represents positive allele from low vigour parent ICC 1882. The fine dotted line from specific linkage group (LG) does not distinguish any parents

Single locus region explained from − 23% to 17% of the phenotypic variation (Additional file 8). For most of the plant vigour traits, the favourable allele was contributed by the high vigour parent ICC 4958, for instance a single locus QTL [13.5% by LG04, 68.09 (AA)] increased PH by 13.5%. Two loci interactions explained from − 23% to 15% of the phenotypic variations (Additional file 8). For instance, two loci interactions [LG07, 63.45 (AA) + LG04, 68.09 (AA)] increased PH by 15% with favourable alleles from ICC 4958. By contrast, two loci interactions [LG04, 99.17 (BB) + LG04, 68.09 (BB)] strongly decreased PHG by − 25% with favourable allele from ICC 1882 (Additional file 8). Three loci interactions explained from − 25% to 17% of the phenotypic variation (Additional file 8). For instance, three loci interactions [LG04, 68.09 (AA) + LG03, 13.00 (AA) + LG03, 3.08 (AA)] increased PH by 17% with favourable allele from high vigour parent ICC 4958. By contrast, three loci interactions [LG08, 51.27 (−) + LG06, 91.97 (BB) + LG04, 24.82 (BB)] increased SLA by 10% with favourable allele from low vigour parent ICC 1882 (Additional file 8 and Fig. 3).

#### Canopy conductance traits

##### QTL analysis for single locus

For TR, one M-QTL (LOD 5 & PVE 10%) was identified in CaLG03 (Additional file 7), and four minor QTL were distributed on CaLG01 (2 QTLs and PVE 5%), CaLG02 (1 QTL and PVE 5%), CaLG07 (1 QTL and PVE 5%; Additional file 7). For eTR, one M-QTL (LOD 6 & PVE 11%) was identified on CaLG04 with favourable allele from ICC 1882. This QTL was located just outside the “*QTL hotspot”* region (Additional file 7). Along with this, four minor QTL were distributed on CaLG04 (2 QTLs & PVE 5-8%) CaLG03 (1 QTL & PVE 8%) and CaLG07 (1 QTL & PVE 5%; see Additional file 7). For T, three minor QTLs were identified, two of these explaining 8-9% phenotypic variation on CaLG04 (“*QTL-hotspot*” region) with favourable allele from ICC 4958. Another one QTL for T was present in CaLG05 (PVE 5%) with favourable allele from ICC 1882 (Additional file 7). For eTR, four minor QTLs were distributed on CaLG04 (2 QTLs & PVE 6-7%), CaLG05 (1 QTL & PVE 5%) and CaLG06 (1 QTL & PVE 5%; Additional file 7).

##### Interaction QTL analysis for multiple loci

E-QTLs interactions identified for canopy conductance (T, TR, eT, eTR & R-3D/PLA) are listed in Additional file 8. Single locus region explained 2.2% to 20% of the phenotypic variation (Additional file 8). For instance, single locus [LG07, 39.00 (BB)] increased R-3D/PLA by 20% with the favourable allele from ICC 1882. Two loci interactions explained from − 43% to 8% of the phenotypic variations (Additional file 8). For instance, two loci interactions [LG07, 37.57 (AA) + LG04, 68.09 (AA)] decreased R-3D/PLA by 43% with favourable allele from ICC 4958. By contrast, two loci interactions [LG07, 12.46 (BB) + LG06, 56.51 (BB)] increased TR by 3.25% with favourable alleles from ICC 1882. Three loci interactions explained from 4% to 31% of the phenotypic variations (Additional file 8). For instance, three loci interactions [LG04, 39.08 (BB) + LG03, 09.09 (BB) + LG01, 16.65 (BB)] increased R-3D/PLA (canopy structure) by 31% with favourable alleles from ICC 1882 (Additional file 8 and Fig. 3). Similarly, a three loci interactions (LG06, 12.48 (BB) + LG04, 44.46 (BB) + LG01, 25.00 (BB)] increased TR by 6.5% with all favourable alleles from ICC 1882 (Additional file 8 and Fig. 3).

#### Co-localization of plant vigour and drought tolerance related traits

**Fig. 4:**
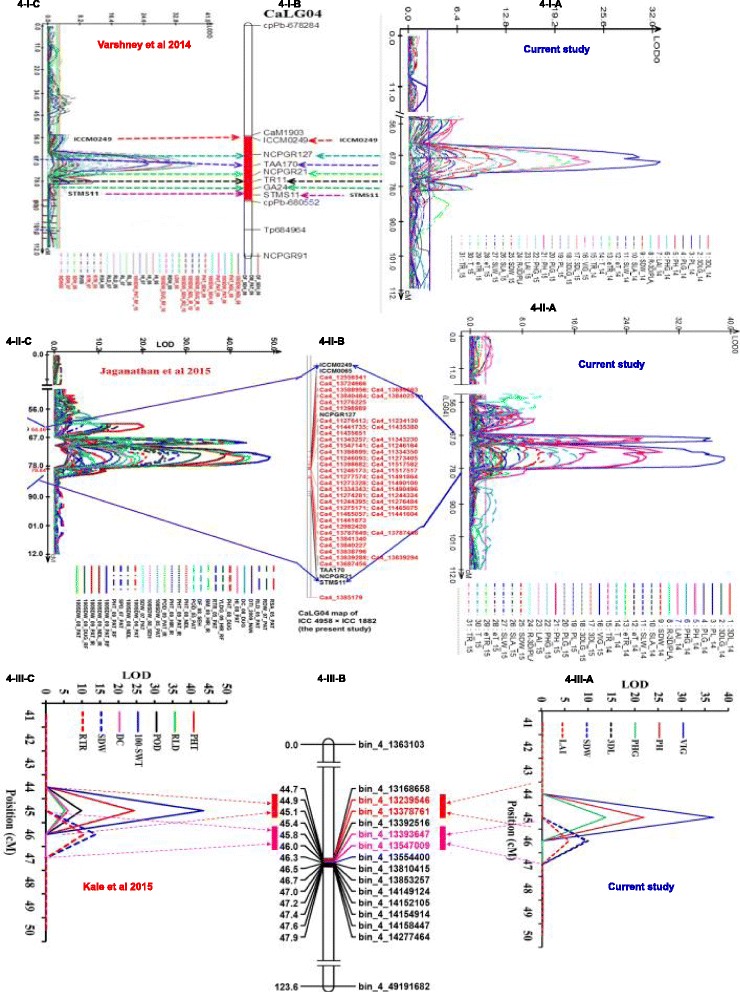
QTL co-localization of plant vigour and drought tolerance related traits using different density markers. Comparison of genomic region with harboring QTLs for various plant vigour and canopy conductance related traits (present study) and drought tolerance traits using 241 SSR-low density marker (Varshney et al. 2014), 1007 SSR + SNP high density marker (Jaganathan et al. 2015) and 1557-SNPs Ultra-high density marker (Kale et al. 2015) identified on CaLG04. The graph 4-I-A, 4-II-A & 4-III-A represent the QTLs identified for various plant vigour and canopy conductance related traits. The graph 4-I-B represent CaLG04 of consensus genetic map; 4-II-B represent CaLG04 of the fine genetic map (Genotype by sequence, GBS approach) and 4-III-B represent CaLG04 of fine bin map (Skim sequencing approach). The graph 4-I-C, 4-II-C & 4-III-C represent QTLs identified for various drought tolerance traits from previous studies. Common QTL regions for both plant vigour and canopy conductance (Present study) and drought tolerance related traits (Varshney et al. 2014; Jaganathan et al. 2015 and Kale et al. 2015) were highlighted in red/pink

Map position of plant vigour traits reported here was compared to map position of roots and drought tolerance traits reported earlier [18, 20, 21]. With the low density marker map, plant vigour traits co-localized with several root traits [eg. root length density, root dry weight/total plant dry weight ratio); see [18] from the previously reported “*QTL-hotspot”* region (Fig. 4-I-A, B & C; Additional file 10).

Similarly, mapping with high density markers data (GBS) showed that plant vigour traits (VIG, 3DL, PL, PH, PHG, LAI and SDW) co-localized with previously identified drought tolerance related traits [roots traits (RLD, RSA, RTR), morphological traits (SDW, PHT, PBS), phenological traits (DF, DM), yield related traits (100 SDW, BM, HI, POD, SPD, YLD) and drought indices (DSI, DTI)] (see, [20]) on CaLG04, which gave also a refined “*QTL hotspot”* region (Fig. 4-II-A, B&C; Additional file 11).

Further co-mapping work was done with the ultra-high density bin marker data (skim sequencing approach). Here, plant vigour related traits (VIG, 3DL, PL, PH, PHG, LAI, SDW) co-localized with previously identified drought tolerance related traits [RLD, RTR%, SDW, PHT, DM, POD, 100SDW, HI and DC; see, [21]] on CaLG04 within the “*QTL- hotspot”* region (Additional file 7; Fig. 4-III-A, B & C).

##### Bin-map “*QTL-hotspot”* region a & b

PH, PHG &VIG had several M-QTLs (LOD 6-37 and PVE 11-53%), and these were identified in the fine mapped “*QTL-hotspot”*-“a*”* region (0.23 cM) on CaLG04. In the same region, PHT, POD, 100-SDW, RLD and DC traits were previously mapped by Kale et al. [21]. Similarly, 3DL, LAI and SDW had several M-QTLs (LOD 5-10 and PVE 11-18%) that were mapped in another “*QTL-hotspot”*-“b*”* region (0.22 cM). In the same region, RTR and SDW traits were previously mapped [21].

#### Asserting QTL location and size in different genetic maps

Different density genetic maps showed QTLs for plant vigour traits on CaLG04 and co-located with the “*QTL-hotspot”* region. Their size within the “*QTL- hotspot”* region using the low density (29 cM size), high density (~ 15 cM size) and ultra-high density maps (“*QTL-hotspot”* a & b (see more details on [21]) together ~300Kb size) on CaLG04 is discussed in this section.

**Fig. 5:**
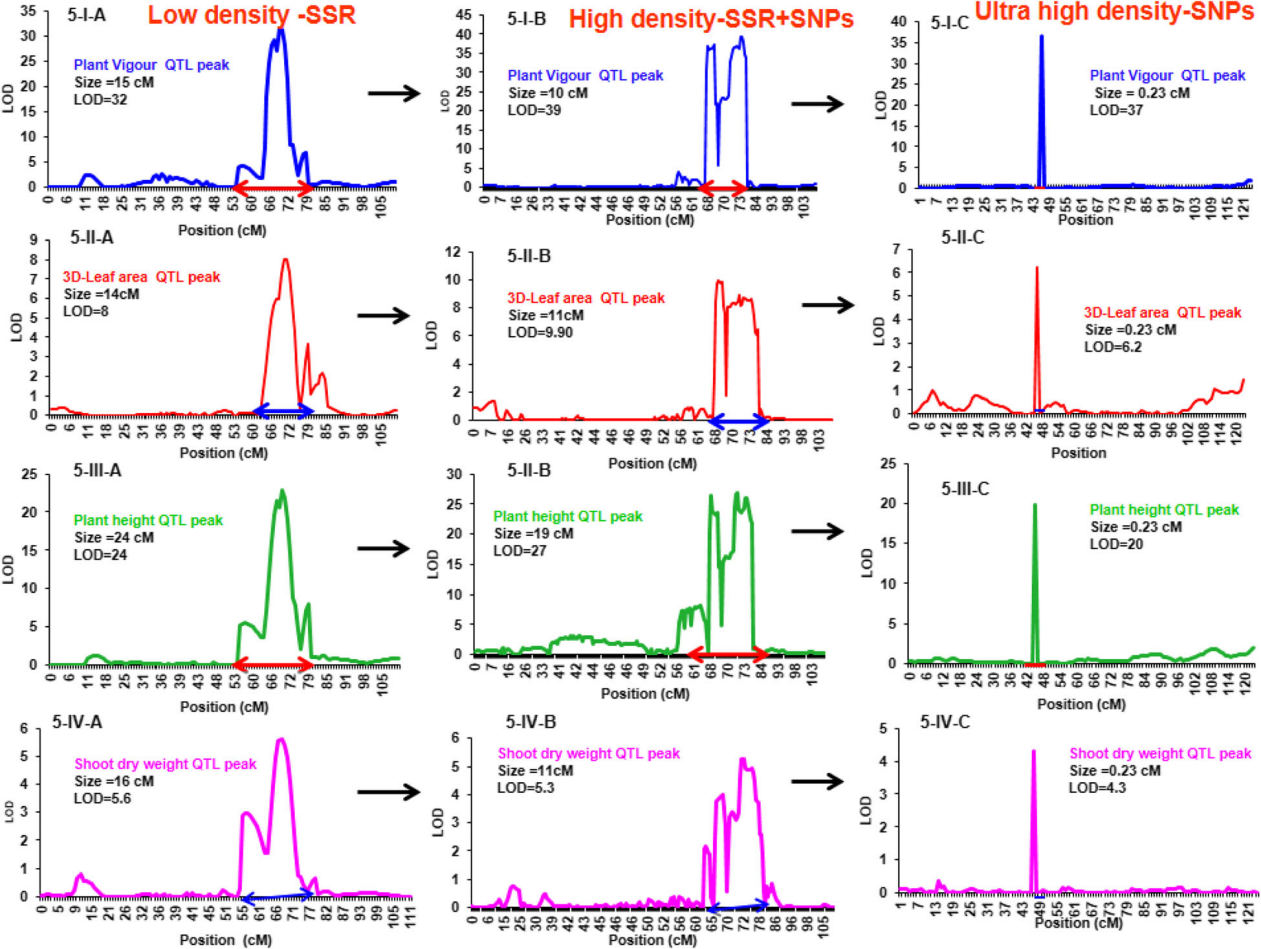
Comparison of M-QTL size for plant vigour related traits using different density markers. Evaluation of M-QTL size performed by using different density markers [A) 241-SSR-Low density marker (Varshney et al. 2014), 1007-SSR + SNPs-high density marker (Jaganathan et al. 2015) and C) 1557-SNPs-Ultra high density (Kale et al. 2015)] on derived mapping population ICC 4958 x ICC 1882. Figure 5-I represent plant vigour QTL peak; 5-II represent 3D-leaf area peak; 5-III represent plant height QTL peak and 5-IV represent shoot dry weight QTL peak

For plant vigour related traits (VIG, 3DL, PL, PH, PHG, LAI, SDW), 28 and 32 M-QTLs were mapped on the low and high density maps, and their size ranged from 1 cM to 8.0 cM on the low density map and 0.8 cM to 5.6 cM on the high density map. For the same traits, the 15 M-QTLs that were mapped using the ultra-high density marker map (Table 3) had a size ranging from 0.14 cM to 0.15 cM. For instance, Fig. 5-I, II, III, IV-A, B&C showed plant vigour traits (VIG, 3DL, PH and SDW) in three different genetic maps. It showed that gradually LOD and PVE increased with marker density and simultaneously QTL size decreased, being fine-tuned down to 300Kb with the ultra- high density marker map. More details on major and minor QTLs for plant vigour in different density genetic maps are presented in Additional files 7, 10 and 11. In addition, low density genetic map along with plant vigour traits QTLs position are shown in Additional file 12A.

**Table 3:** Summary of Major-QTLs (M-QTLs) for plant vigour and canopy conductance related traits using different genetic map. Low density (241 SSR marker-Varshney et al. 2014); high density (1007 SSR + SNP marker- Jaganathan et al. 2015) and ultra-high density (1557 SNP markers- Kale et al. 2015) markers were used for identification of QTLs. The trait on only measured at 2015 indicates (+) and newly identified additional QTLs with high density markers were indicated by (*). Details of traits code were mentioned in Table 1

Marker used	Trait code	Linkage groups (LGs)	Total QTLs	No. of QTLs in the QTL hotspot	Consistent QTLs	Genetic Size (cM)	Logarithm of the odds ratio (LOD)	Phenotypic variation explained (PVE, %)
Low density-SSR	VIG	4	2	2	+	2.00	7.0-32	13-44
High density-SSR + SNPs	VIG	4	2	2	+	0.4-2.7	36-39	47-51
Ultra-high density-SNPs	VIG	4	1	1	+	0.14	36.7	53.00
Low density-SSR	3DL	4	5	5	2	1.0-6.0	5.0-12	10-23
High density-SSR + SNPs	3DL	4	5	5	3	0.4-3.6	6.0-13	11-20
Ultra-high density-SNPs	3DL	4&6	4	3	1	0.15-13	2.3-9.8	11-19
Low density-SSR	PL	4	3	3	1	5.0-7.0	6.0-6.0	12-13
High density-SSR + SNPs	PL	4	3	3	1	1.3-5.6	6.0-9.0	10-14
Ultra-high density-SNPs	PL	4	1	1	1	0.05	5.6	11
Low density-SSR	SDW	4	5	5	3	3.0-7.0	4.0-10	10-20
High density-SSR + SNPs	SDW	4	6*	6	3	0.9-2.8	5.0-11	11-18
Ultra-high density-SNPs	SDW	4	1	1	1	0.15	9.3	18
Low density-SSR	LAI	4	2	2	2	4.0-7.0	5.0-7.0	10-16
High density-SSR + SNPs	LAI	4	1	1	–	0.8	6.0	10
Ultra-high density-SNPs	LAI	4	1	1	1	0.15	5.7	11
Low density-SSR	PH	4	6	6	2	2.0-8.0	6.0-23	10-32
High density-SSR + SNPs	PH	4	6	6	2	0.8-2.9	8.0-29	14-37
Ultra-high density-SNPs	PH	2,4&7	7*	5	3	3.4-0.14-0.10	4.9-21.7	10-39
Low density-SSR	PHG	4	5	5	2	3.0-4.0	5.0-13	11-25
High density-SSR + SNPs	PHG	4	9*	9	3	1.1-4.6	7.0-17	
Ultra-high density-SNPs	PHG	4&7	4	3	1	0.14-0.07	4.8-13.6	10-23
Low density-SSR	eT	4	1	1	1	8.0	5.0	11
High density-SSR + SNPs	eT	4	1	1	1	0.21	4.0	12
Ultra-high density-SNPs	eT	–	–	–	–	–	–	–
Low density-SSR	eTR	4	2	–	1	7.0-10	6.0-8.0	10-11
High density-SSR + SNPs	eTR	3&4	4*	3	2	2.0-5.0	3.0-6.0	11-14
Ultra-high density-SNPs	eTR	4	1	–	1	0.48	5.7	11
Low density-SSR	T	4&8	2	1	1	6.0-8.0	5.0	12-14
High density-SSR + SNPs	T	5&8	2	–	–	2.9-3.5	3.0-6.0	10-14
Ultra-high density-SNPs	T	–	–	–	–	–	–	–
Low density-SSR	TR	7	3	–	1	5.0-13	3.0-5.0	10-17
High density-SSR + SNPs	TR	–	–	–	–	–	–	–
Ultra-high density-SNPs	TR	3	1	–	–	0.08	5.1	10
Low density-SSR	R-3D/PLA	4, 6 &7	10	–	5	1.0-15.0	6.0-13	10-15
High density-SSR + SNPs	R-3D/PLA	1,4, 6&7	13*	1	4	0.3-4.2	7.0-14	10-16
Ultra-high density-SNPs	R-3D/PLA		–	–	–	–	–	–

For canopy conductance traits (TR, eTR, T, eT and R-3D/PLA), several QTLs were identified on different linkage groups (CaLG01, LG03, LG04, LG05, LG06, LG07 & LG08) across the genome. A total of 18 and 20 M-QTLs were mapped on different LGs using low and high density maps, respectively (see more details in Additional files 10 and 11). The QTL size ranged from 1 cM to 15.0 cM size in low density map and high density ranged from 0.3 cM to 5.0 cM size (Table 3). Two M-QTLs were mapped on CaLG03 (TR) and just outside the CaLG04 “*QTL-hotspot”* region (eTR) using ultra-high density map. The QTL size ranged from 0.08 cM (TR) to 0.48 cM (eTR) size (Table 3). For TR, three M-QTLs with 5-13 cM were identified on CaLG07 using low density marker (Table 3). In the high density marker, no M-QTL was detected for TR. But, six minor QTLs were identified on CaLG03 (2QTLs; 4.9-5.1 cM), CaLG07 (1QTL; 2.0 cM), CaLG06 (1QTL; 10.3 cM) and CaLG04 (2.3-11.9 cM; Additional file 11). On the ultra-high density map, one M-QTL for TR was mapped on CaLG03 (0.08 cM). For TR, mapping position varied between low and ultra-high density markers. This might be most of the similar alleles between CaLG03 and CaLG07 (Table 3). Similarly for R-3D/PLA (canopy structure), 10 M-QTLs were identified on different linkage groups [CaLG04 (7 M-QTLs; 1.0-8.0 cM), CaLG06 (2 M-QTLs; 11-15 cM) & CaLG07 (1 M-QTL; 2.0 cM)] using low density markers (Table 3 & see more details in Additional file 10). For high density markers, 13 M-QTLs were identified on different linkage groups [CaLG04 (7 M-QTLs; 0.3-1.3 cM), CaLG06 (2 M-QTLs; 2.6-4.2 cM), CaLG07 (2 M-QTLs; 2.4-2.6 cM) and CaLG01 (2 M-QTLs; 4.1-4.2 cM)] (Table 3 & see more details in Additional file 11). There was no QTL was detected with the ultra-high density markers map (Table 3). More details of canopy conductance traits major and minor QTLs in different density genetic maps are presented in Additional files 7, 10 and 11. In addition, low density genetic map along with canopy conductance traits QTLs position is shown in Additional file 12B, C & D.

## Discussion

The summary of the main results is as follows: i) Genetic variation of 16 phenotypic traits revealed two clusters of plant vigour and canopy conductance traits and their association was clarified with PCA analysis and correlation. ii) Using the ultra-high density map, M-QTLs for plant vigour traits predominantly mapped on CaLG04 and these co-mapped with a previously refined *“QTL-hotspot”* region (~300Kb) for drought tolerance traits. Canopy conductance traits were mapped in CaLG03 (TR) and CaLG04 (eTR). iii) The refined *“QTL-hotspot”* region (Bin-Map) was further sub-divided into a *“QTL-hotspot*- a*”* and *“QTL-hotspot*- b*”* regions. While both *“QTL-hotspot”* sub-regions co-mapped with previous study [21], the phenotyping data at a lower level of plant organization gathered here led us to interpret that region ‘a’ (139.22Kb or 0.23 cM) could be a locus for branching and tissue/organ expansive processes while region ‘b’ (153.36Kb or 0.22 cM) could be interpreted as a locus for physiological processes related to biomass accumulation. iv) As marker density increased QTL number and size decreased (~ 29 cM to 0.22 cM); and LOD and PVE (%) increased for most of the QTLs. v) Most of plant vigour traits had alleles from high vigour parent ICC 4958 whereas in the case of canopy conductance traits (eTR and TR) the favourable alleles were contributed by the low vigour parent ICC 1882. vi) Plant vigour traits mapped mostly on CaLG04 whereas canopy conductance traits mapped on CaLG03, providing an opportunity to manipulate these loci to tailor recombinants having lower transpiration rate and high plant vigour desirable for water limited environments.

### Phenotyping at different level of plant organization

The vigour traits (3DL, PL, SDW, PH, and 3D-LG) were tightly linked to plant water use traits. These traits were reported to be linked to crop biomass production and then crop yield [25, 26]. The co-localization study clearly demonstrated the close relationship between traits from the present study at a lower level of plant organization (eg. 3D-Leaf area, growth rate) and the agronomic traits (eg.shoot biomass, harvest index) studied previously by Varshney et al. [18]. Canopy development traits had also a clear effect on crop production [25]. Although phenotyping of traits at a lower level of plant organization is usually laborious and time-consuming process, it was facilitated by the use of a high throughput phenotyping platform (LeasyScan). Most of the plant vigour traits had high heritabilities, making them suitable for breeding applications. The high vigour parent ICC 4958 had higher biomass and water use (absolute T) than the low vigour parent ICC 1882. By contrast, the high vigour parent had lower transpiration rate (TR; g of water transpired per unit of leaf area) than low vigour parent. Hence, the cause for such response in water-use was the difference in leaf area (vigour/canopy development). The effect of such combination, having high vigour and lower TR would be then of high value to test across time and geographical scale using crop simulation analysis. Crop simulation modelling of water saving traits (eg. limited transpiration rate) has indeed shown a clear yield advantage under terminal drought stress conditions (Soybean-[27], Maize-[28] and Sorghum-[15]).

### Co-localization of plant vigour traits and previously identified drought tolerance traits in different genetic maps

Early plant vigour is an important trait for water limited environments. It may contribute to shading of the soil surface, thereby reducing evaporation of water from the soil and leaving more water available for the crop [25, 29, 30]. In the present study, most of the plant vigour traits had several M-QTLs on CaLG04 and co-mapped with the earlier reported fine mapped “*QTL hotspot”* region [18, 20, 21] with QTLs for root traits. The alleles for these vigour traits were contributed by high vigour parent ICC 4958. Here is a first detailed study reporting the co-mapping of plant vigour traits with root and so-called drought tolerance traits. This is also a confirmation of the earlier observations that shoot dry weight and root length density QTL co-mapping in preliminary results [22]. This result, therefore, suggests that the drought tolerance reported earlier to be associated with that QTL in the hotspot region (241 Low density SSR marker-[18]; 1007-High density GBS markers-[20] and Ultra-high density Bin maps-[21]) would actually be conferred by plant vigour aspects. Such result was also predicted by a crop simulation study [31] that concluded that in the short duration environments where chickpea cultivation is now mostly cultivated, a high plant vigour associated with faster rooting would be necessary to reach the water available deep in the soil profile. Similarly, in recent pearl millet mapping studies [32, 33] it was reported that plant vigour traits also co-localized with agronomic traits related to terminal drought tolerance [34]; drought index of stover yield, grain yield, biomass yield and harvest index [35–37]. Similarly, another study in a high- resolution cross (HRC) population of pearl millet showed that plant vigour traits (3D-leaf area, plant growth rate, plant height) measured from LeasyScan co-localized with yield traits measured in the field under different water stress treatments (Tharanya et al-unpublished data. The present study suggests that high root length density obtained earlier [18] could be more easily proxied by vigour traits at the canopy level, which would then ease the phenotyping of that particular trait. Overall, plant vigour traits might lead to high biomass, which would then link to higher yield potential. Therefore, the genotypes that have alleles from ICC 4958 would be beneficial for water limited conditions.

#### Binmap QTL hotspot region

With the ultra-high density marker, the refined *QTL-hotspot* was sub-divided into two sub-regions “*QTL-hotspot”-“a”* & “*QTL-hotspot”-“b”*. Our interpretation, on the basis of the phenotyping at a lower level of plant organization done in the present study, is that these two regions could control two domains of physiological processes. “*QTL-hotspot”-“a”* region, which had QTL for traits related to vigour and growth rate (PH and VIG), could be interpreted as a region coding for branching and expansive processes. We interpret the possible effect on the branching from the two fairly opposite phenotypes of the parents of the population used here, i.e. highly branched ICC 1882 with low height versus less branched but taller ICC 4958. More work would be needed to decipher in more details the possible interaction between height and branching. The interpretation of the expansive processes comes from recent genetic work on regions controlling leaf expansion in maize [38], and where vigour could simply be consequences of differences in the expansive processes leading to larger organ sizes and quicker development. Interestingly, this region ‘a’ was earlier reported to harbour QTL for pod number per plant, 100-seed weight and plant height [21], although a finer analysis of the plant processes possibly involved was not done. It was particularly interesting to see that this region led to seed size differences, which then raises the question whether seed size is not itself controlled by expansive processes at the time of embryo development and seed formation. “*QTL-hotspot”-“b”* region could then simply be a locus controlling physiological processes involved in biomass accumulation, which was corroborated by the QTLs found here for 3DL, LAI and SDW, or for biomass traits SDW and RTR traits in Kale et al. [21].

Twelve candidate genes were reported from this fine mapped “*QTL hotspot”* region (see, [21]) stating that most of the genes were involved in abiotic stress tolerance. The same genes were also reported to be associated with plant growth and development related functions (e.g. genes of serine threonine-protein kinases, E3 ubiquitin ligases, Leucine-rich repeat extension (LRXs), Protein IQ domain and Vicilin 47 K and Cotyledon vascular pattern (CVP2) genes that were reported to be associated with drought stress adaptation by Kale et al., [21] were also reported to be associated with plant growth and development related process [39–48]. These reports additionally suggest that earlier reported “*QTL-hotspot”* region to be associated more likely with vigour related traits.

### Ideotyping of plant vigour and canopy conductance genomic regions

An ideal ideotype for water limited environment would be the one having higher plant vigour (the proxy for higher biomass and yield) potential with restriction of transpiration under high VPD conditions. These combinations would achieve higher water use efficiency, eventually soil moisture conservation, and then ultimately lead to crop production success. The plant vigour traits were mapped on CaLG04 and the canopy conductance (eg. TR) traits were present on CaLG03. These two genomic regions contributed more than 75% QTLs for plant water use (vigour and conductance) traits. Therefore, CaLG04 (plant vigour) and CaLG03 (canopy conductance) provide an opportunity to manipulate these loci to tailor recombinants having alleles with lower transpiration rate along with high plant vigour. This ideotype might be useful in enhancing the water stress adaptation in chickpea. Similar kind of ideotyping was recommended in pearl millet [32, 33]. Recent modelling reports on sorghum [15] showed that alteration of leaf area (plant vigour components) and transpiration rate increased grain yield under severe stress conditions. This study suggests that plant vigour and transpiration rate trait assessed in the current study might also have an effect on crop production success in specific target environments.

## Conclusion

The present study has shown that a previously identified “QTL hotspot” region on LG04 of chickpea and harbouring QTL for root traits and so-called terminal drought tolerance in chickpea was a vigor locus, with favourable alleles from high vigour parent ICC 4958. Our phenotypic analysis at a lower level of plant organization led us to interpret that this locus may be divided into two sub-regions, one coding for expansive processes and one for biomass accumulation. Another genomic region on CaLG03 harboured QTL for canopy conductance traits (e.g. TR). Plant vigour and canopy conductance traits were somewhat negatively related but being mapped on different chromosome provides an opportunity to manipulate these loci to tailor recombinants having lower transpiration rate and high plant vigour which would be useful enhancing the drought adaptation in chickpea. In addition, potential genomic region on CaLG04 with simple vigour traits (e.g vigour score) could be used for breeding programs through marker assisted backcross (MAB) to devolep improved variety. Enrichment of the marker density reduced QTL size and increased in LOD and PVE% for all plant vigour and canopy conductance traits.

## Methods

### Plant material

The genetic material was a set of 232 recombinant inbred lines from a population derived by single seed descent method from the cross between ICC 4958 and ICC 1882 and advanced to F10+ generation [18]. Genotype ICC 4958 is a drought tolerant breeding line developed by Jawaharlal Nehru Krishi Vishwa Vidyalaya, Jabalpur, and Madhya Pradesh, India. It has a large root system, early vigour is early to reach 50% of flowering (608 cumulative degrees) and maturity (1650 cumulative degrees). The ICC 1882 landrace was collected in India and added to the ICRISAT’s genebank in 1973. It has a small root system, late vigour, is later to reach 50% of flowering (779 cumulative degrees) and maturity (1806 cumulative degrees) compared to ICC 4958 [8, 18, 49]. These two parental lines were contrasting for root traits and plant vigour i.e. were used for mapping population development. Additional detail account on parental lines and mapping population are provided in Varshney et al. [18].

### Crop Phenotyping

#### Plant growth conditions

Phenotyping was performed from November to December 2014 & 2015 in the LeasyScan facility [23]. Plants were sown during the post-rainy chickpea sowing window (November). Plants were grown in 27 cm diameter plastic pots filled initially with 9 kg of dry black soil (Vertisol) collected from ICRISAT farm. Each experimental unit in the LeasyScan platform was composed of 2 pots, each containing 4 healthy plants. These experimental units being of 65 × 40 cm, i.e. approximately 0.25 m^2^, the sowing density was 32 plant m^− 2^, which is equivalent to the sowing density in the field. In other words, phenotyping was done on a crop canopy that had close similarities with a field situation. Sowing was done with 6-8 seeds per pot and seedlings were thinned to maintain four homogeneous seedlings per pot at 12 days after sowing (DAS). Fertilizers were provided with single super phosphate (SSP) as basal dose at the rate of 0.3 g/kg of soil. The experimental design was an Alpha lattice with 4 replications and 24 blocks of 10 genotypes in each replication to avoid geographical variations. Plants were maintained under well water conditions the throughout experiment. During the crop grown period, 11/35.8 °C minimum and maximum temperature and 17.2/93.2% relative humidity were observed.

### Phenotypic traits evaluated

Sixteen phenotypic traits were measured and categorized into three groups: (i) Canopy traits (measured by LeasyScan) (ii) Transpiration traits (measured by gravimetric balance system) and (iii) Biomass traits.

#### i) Canopy traits

LeasyScan PlantEye^®^ scanners measured canopy development related traits [3DLeaf area (3D-L), projected leaf area (PL) and plant height (PH)] on the hourly basis during crop growth periods. Using these traits, plant growth rate related traits [3D-Leaf area growth rate (3D-LG), projected leaf area growth rate (PLG), plant height growth rate (PHG)] were calculated. Plant growth rate (3DLG, PLG, PHG) was calculated based on the average difference in respective leaf area and plant height between consecutive days during the exponential growth phase. The leaf area index (LAI) was estimated as the projected leaf area PL divided by the area of the pots in the sector. Plant vigour score was estimated by visual eye basis, on a scale from 1 (low vigour) to 5 (high vigour) at 20 DAS after sowing, all four replications being scored by one person eye visual score. Similar protocal was reported in other crop species such as wheat [50] and maize [51]. Residual (canopy structure) was calculated by using 3D-leaf area and projected leaf area.

#### ii) Transpiration traits

Transpiration (evapotranspiration (eT)) was measured by a gravimetric method (see [32]). The pots were watered abundantly and drained overnight to attain field capacity. An extra 20 pots without plants were also brought to field capacity and were there to evaluate soil evaporation. Following day, plants were manually weighed (Model FCB 24 K0.2B, KERN & Sohn GmbH, Baligen, Germany.). All four replications were weighed between 6 and 7 am (Initial weight; average VPD~ 0.8 kPa). Pots were weighed again late afternoon between 3 and 4 pm (final weight; average VPD ~ 3.76 kPa), following the same sequence of pot weighing as in the morning. Evapotranspiration was calculated by the difference between initial and final pot weight. Further, plant transpiration (T) was estimated by subtracting an estimate of soil evaporation (pot without plant soil evaporation). Briefly, it was assumed that soil evaporation in planted pot would be maximum with zero plant cover, and would be zero at a leaf area index of 2.

Therefore, the projected leaf area was used to infer a LAI. Briefly,

LAI = PL/area of the pots in the sector.

At the time of eT measurements and transpiration values were estimated from this correction. While this may have induced some error, we made the assumption the method would be correct for genotypic comparison and QTL analysis. Transpiration rate (TR) and evapotranspiration rate (eTR) were calculated by transpiration and evapotranspiration divided by 3D-leaf area and time [52].

#### iii) Biomass traits

At the end of the experiment (canopy covered maximum in the pot; 35 DAS), shoot samples were harvested and over dried at 65 °C for 48 h. Further, shoot dry weights (SDW) were weighed using gravimetric balance (KERN 3Kg) method. Specific leaf area (SLA) was estimated by leaf area divided by shoot dry weight. Specific leaf weight (SLW) was estimated by 1/SLA (inverse of SLA).

#### QTL analysis- single locus

QTL analysis was conducted independently using three genetic maps developed earlier [18, 20, 21] and phenotyping data generated in this study. QTL Cartographer version 2.5, composite interval mapping (CIM) method was employed [53]. For ultra-high density bin markers, inclusive composite interval mapping-Additive mapping (ICIM-ADD) method was used for identification of QTLs using IciMapping software (v3.2; [54]). LOD threshold was set by using 1000 permutation and *p* value ≤0.05. Constructed linkage map was visualized using Mapchart 2.2 [55] software. When the PVE (phenotypic variation explained) was above 10%, QTLs were considered major QTLs (M-QTLs) and PVE below 10% were minor QTLs.

#### Interactions QTL analysis-multi-loci

The QTL interactions influencing the traits were identified using Genotype Matrix Mapping software (GMM; v. 2.1; [56], http://www.kazusa.or.jp/GMM). Using GMM, two and three loci interactions were tested. GMM analysis showed interactions between loci and different linkage groups of plant vigour and canopy conductance related traits. The current study identified allelic interactions that contributed to either a positive (increase) or negative (decrease) effect on the phenotypic value of the trait. In most cases, single locus QTL identified using GMM analysis were similar to those identified with CIM analysis, even though two approaches use different algorithms. In the following text, symbols “AA”, “BB” and “stand for alleles originated from the high vigour parent (AA; ICC 4958) and low vigour parent (BB; ICC 1882)” and not distinguished from any parent (−), respectively.

#### Statistical analysis

To find the phenotypic variations and their significance in the population, ANOVA was performed for all observed parameters individually using GENSTAT 14.0 (VSN International Ltd., Hemel Hempstead, UK). Similarly, to find the phenotypic variations and their significance in parental lines were analyzed with statistical program package CoStat version 6.204 (Cohort Software, Monterey, CA, USA). One-way ANOVA was carried out to test for genotypic difference between the genotypes. Means were compared using Tukey-Kramer test and Least Significant Difference (at *P* ≤ 0.05). Normal histograms with frequency distribution analysis for phenotypic traits were done using SPSS 16 desktop version (IBM, SPSS Statistical software). Principal component analysis (PCA) was used to visualize the relationships between traits in a multidimensional space using R software (version 2.11.1). To find the trait correlation of all phenotypic traits, simple Pearson correlation was performed using R software (version 2.11.1). For QTL and PCA analysis, Best Linear Unbiased Predictors (BLUPs) data were estimated by using GENSTAT 14.0 were used. The clustering analysis was performed by PCA loadings using R software (version 2.11.1). Genotypic and residuals mean square components were obtained from ANOVA through GENSTAT 14.0, which was used to calculate the broad sense heritability (*h*^*2*^). The broad-sense heritability (*h*^*2*^) was calculated as *h*^2^ = σ ^2^ G/ (σ ^2^ G + σ ^2^ E) [31, 32], where σ ^2^ G is the genetic variance and σ ^2^ E is the error variance.

## Additional files

Additional file 1: Cluster dendrogram analysis for collected phenotypic traits. Cluster analysis performed by major principal components using R-package. Two clusters (1 and 2) were shown on plant vigour traits and canopy conductance traits. (PPTX 55 kb)

Additional file 2: Frequency distribution of plant vigour and canopy conductance related traits. Frequency distribution of plant vigour (A, B, C & D) and canopy conductance (E, F, G & H) related traits in chickpea mapping population (ICC 4958 x ICC 1882) showing normal distribution. A, B, C & D represent the plant vigour, plant height, 3D-leaf area and shoot dry weight (Plant vigour related traits) and E, F, G & H represent transpiration rate, evapotranspiration rate, transpiration and evapotranspiration (Canopy conductance related traits). P1 and P2 represent the ICC 4958 and ICC 1882). (PPTX 220 kb)

Additional file 3: Growth dynamics of canopy development in contrasting parental lines. Growth dynamics of A) 3-leaf area and B) plant height in contrasting parental lines [High vigour parent (ICC 4958) and low vigour parent (ICC 1882) at vegetative stage calculated on the basis of thermal time (228-806 degree days for A and 114-806 degree days for B). (PPTX 181 kb)

Additional file 4: Trait correlation analysis for the plant vigour and canopy conductance related traits. All the traits were evaluated under high throughput plant phenotyping platform (LeasyScan). (XLSX 10 kb)

Additional file 5: Graphical representation of principal component analysis (PCA) for plant vigour and canopy conductance related traits. The plant vigour and canopy conductance traits vectors are represented by red arrows. The numbers represent recombinant inbred lines numbers (RIL numbers) and its position represents the particular trait loadings with respect to PC1 and PC2. BLUPs data across years were used for PCA analysis. (PPTX 117 kb)

Additional file 6 Details on principal component analysis (PCA) for plant vigour and canopy conductance traits. (XLSX 10 kb)

Additional file 7: Summary of QTLs for plant vigour and canopy conductance related traits using ultra-high density map. The QTLs were identified using ICIM (QTL IciMapping) software on ICC 4958 × ICC 1882 derived mapping population. (XLSX 12 kb)

Additional file 8: Selected E-QTL interactions for plant vigour and canopy conductance traits. The E-QTLs were identified using genotype matrix mapping (GMM) software on ICC 4958 × ICC 1882 derived mapping population. (XLSX 12 kb)

Additional file 9: Summary of E-QTL interactions for plant vigour and canopy conductance traits. The E-QTLs were identified using genotype matrix mapping (GMM) software on ICC 4958 × ICC 1882 derived mapping population. (XLSX 24 kb)

Additional file 10: Summary of QTLs for plant vigour and canopy conductance related traits using low density marker. The QTLs were identified using QTL Cartographer software on ICC 4958 × ICC 1882 derived mapping population. (XLSX 17 kb)

Additional file 11: Summary of QTLs for plant vigour and canopy conductance related traits using high density marker. The QTLs were identified using QTL Cartographer software on ICC 4958 × ICC 1882 derived mapping population. (XLSX 20 kb)

Additional file 12: Genetic map of chickpea RIL population derived from ICC 4958 x ICC 1882. The genetic map represents marker position and corresponding marker name in linkage group. Genetic distances (cM) were shown on the left and markers are shown on the right side of the bars. The map was constructed using Map chart software. The Q represents QTL and R3 represents the population name. Year of mapping was represented by the symbols: # (2014), + (2015) and $ (across the year). **A** represent CaLG04 with identified QTLs position and its corresponding marker. The markers of the QTLs regions within the hotspot (most plant vigour related traits) were represented in red and outside the hotspot were represented in pink (QR3R-3D/PLA # + $), green (QR3-eTR+ $) and brown (QR3R-3D/PLA $). **B)** Map represents CaLG07 with QTLs for transpiration rate (QR3-TR) (markers highlighted in red colour) and residuals from 3D and projected leaf area (QR3R-3D/PLA) (markers highlighted in pink colour). **C)** Map represents CaLG06 with QTLs for residuals from 3D and projected leaf area (QR3R-3D/PLA) (markers highlighted in red colour). **D)** Map represents CaLG08 with QTLs for transpiration (QR3-T) (markers highlighted in red colour). (ZIP 283 kb)

## Abbreviations

ANOVA: Analysis of variance
BLUPs: Best linear unbiased predictions
CIM: Composite interval mapping
DAS: Days after sowing
E-QTLs: Epistatic QTLs
GBS: Genotype by sequencing
GMM: Genotype matrix mapping
h^2^: Heritability
ICIM-ADD: Inclusive composite interval mapping-Additive mapping
LG: Linkage group
LOD: Logarithm of odds
LSD: Least significant difference
M-QTLs: Major QTLs
PCA: Principal component analysis
PVE: Phenotypic variation explained
QTL: Quantitative trait loci
RILs: Recombinant inbred lines
SNPs: Single nucleotide polymorphisms
SSRs: Simple sequence repeats
