# Supplementary figures and images for: Plant vigour QTLs co-map with an earlier reported QTL hotspot for drought tolerance while water saving QTLs map in other regions of the chickpea genome
Source: BMC Plant Biol. 2018 Feb 6;18:29. doi: 10.1186/s12870-018-1245-1 (PMC5801699; doi:10.1186/s12870-018-1245-1)

## Slide 1
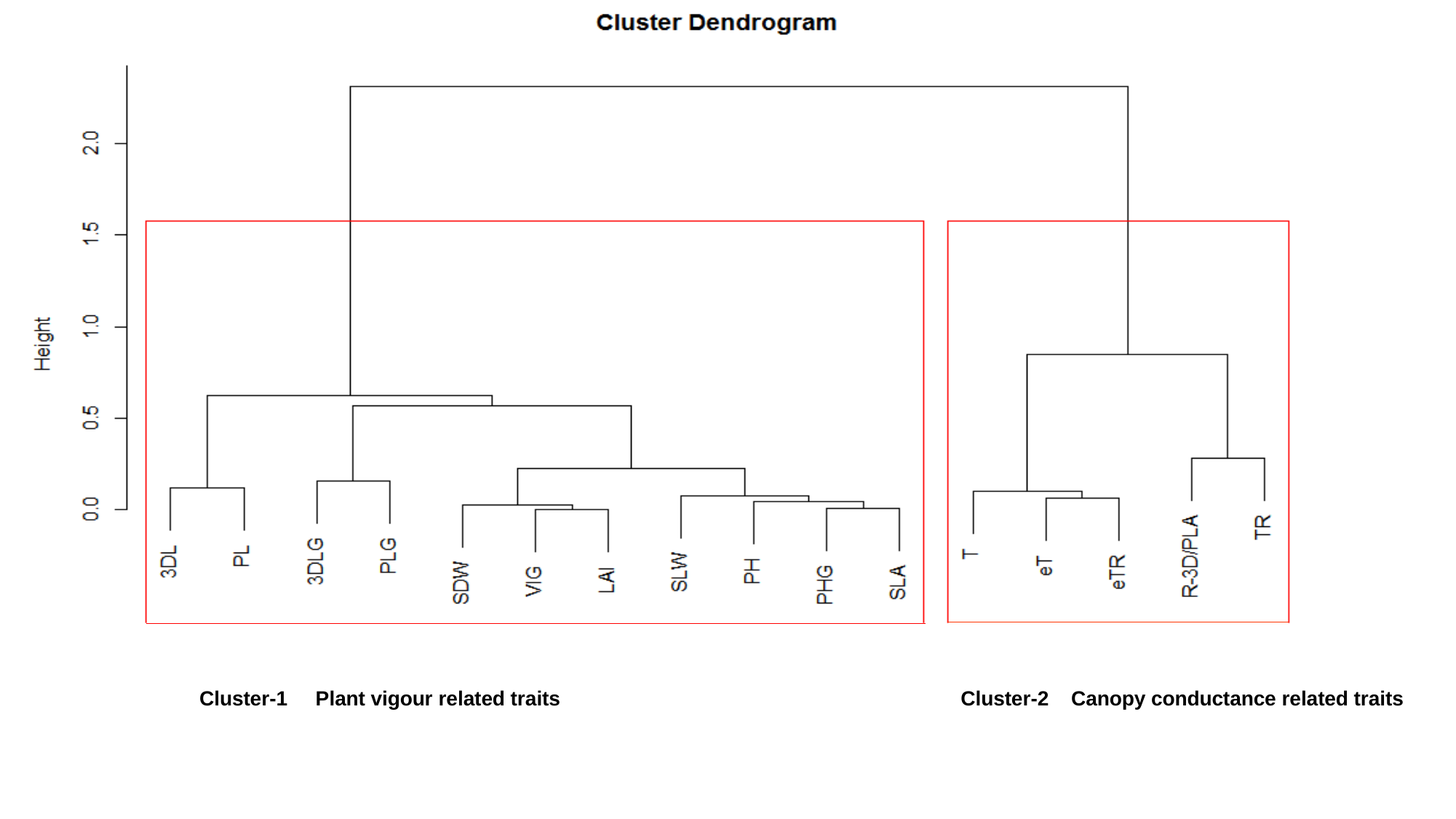

Cluster-1 Plant vigour related traits
Cluster-2 Canopy conductance related traits

Supplement: Supplementary file 1 — Cluster dendrogram analysis for collected phenotypic traits. Cluster analysis performed by major principal components using R-package. Two clusters (1 and 2) were shown on plant vigour traits and canopy conductance traits. (PPTX 55 kb) [file 12870_2018_1245_MOESM1_ESM.pptx]

## Slide 1
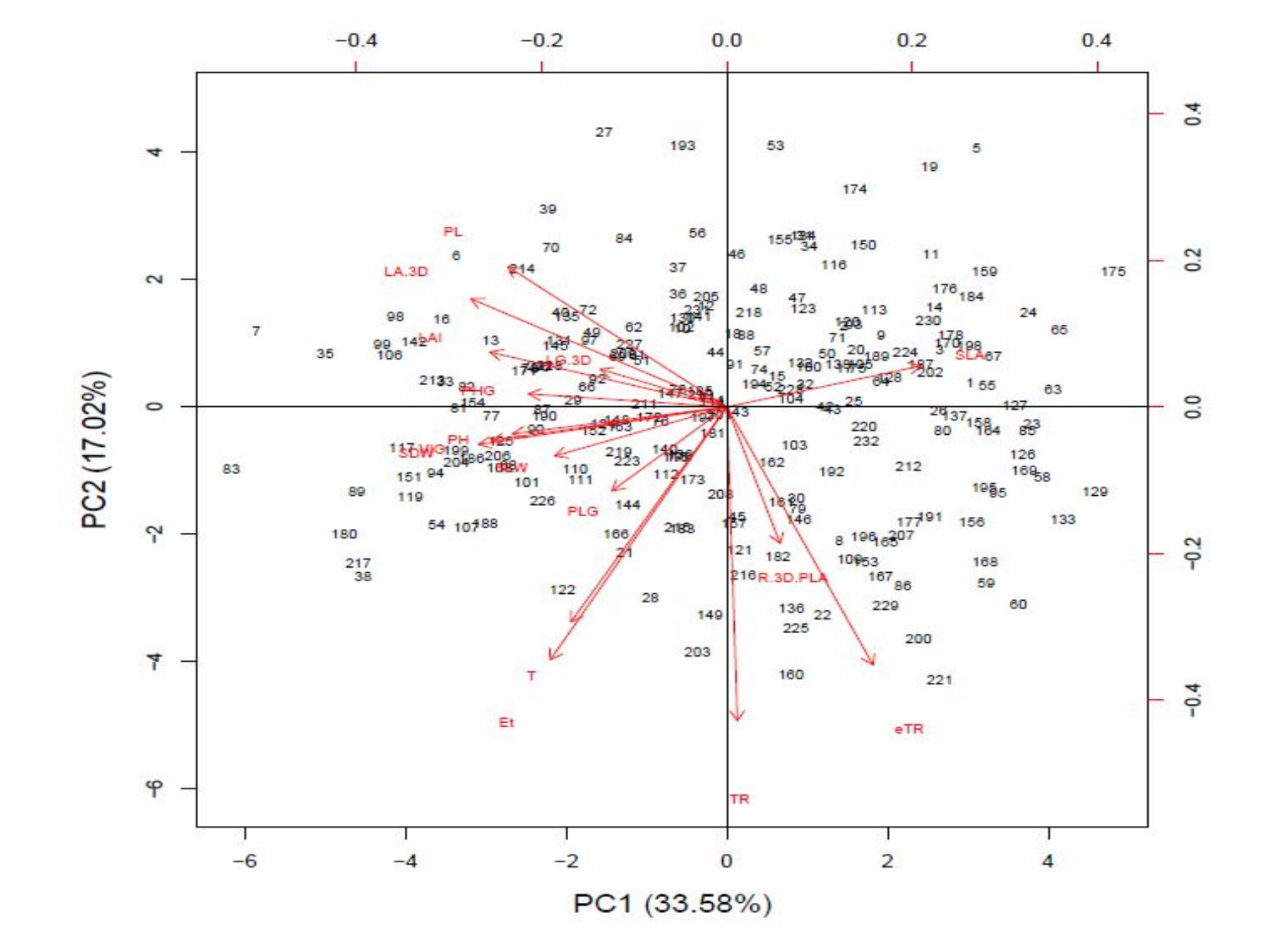

Supplement: Supplementary file 5 — Graphical representation of principal component analysis (PCA) for plant vigour and canopy conductance related traits. The plant vigour and canopy conductance traits vectors are represented by red arrows. The numbers represent recombinant inbred lines numbers (RIL numbers) and its position represents the particular trait loadings with respect to PC1 and PC2. BLUPs data across years were used for PCA analysis. (PPTX 117 kb) [file 12870_2018_1245_MOESM5_ESM.pptx]

## Slide 1
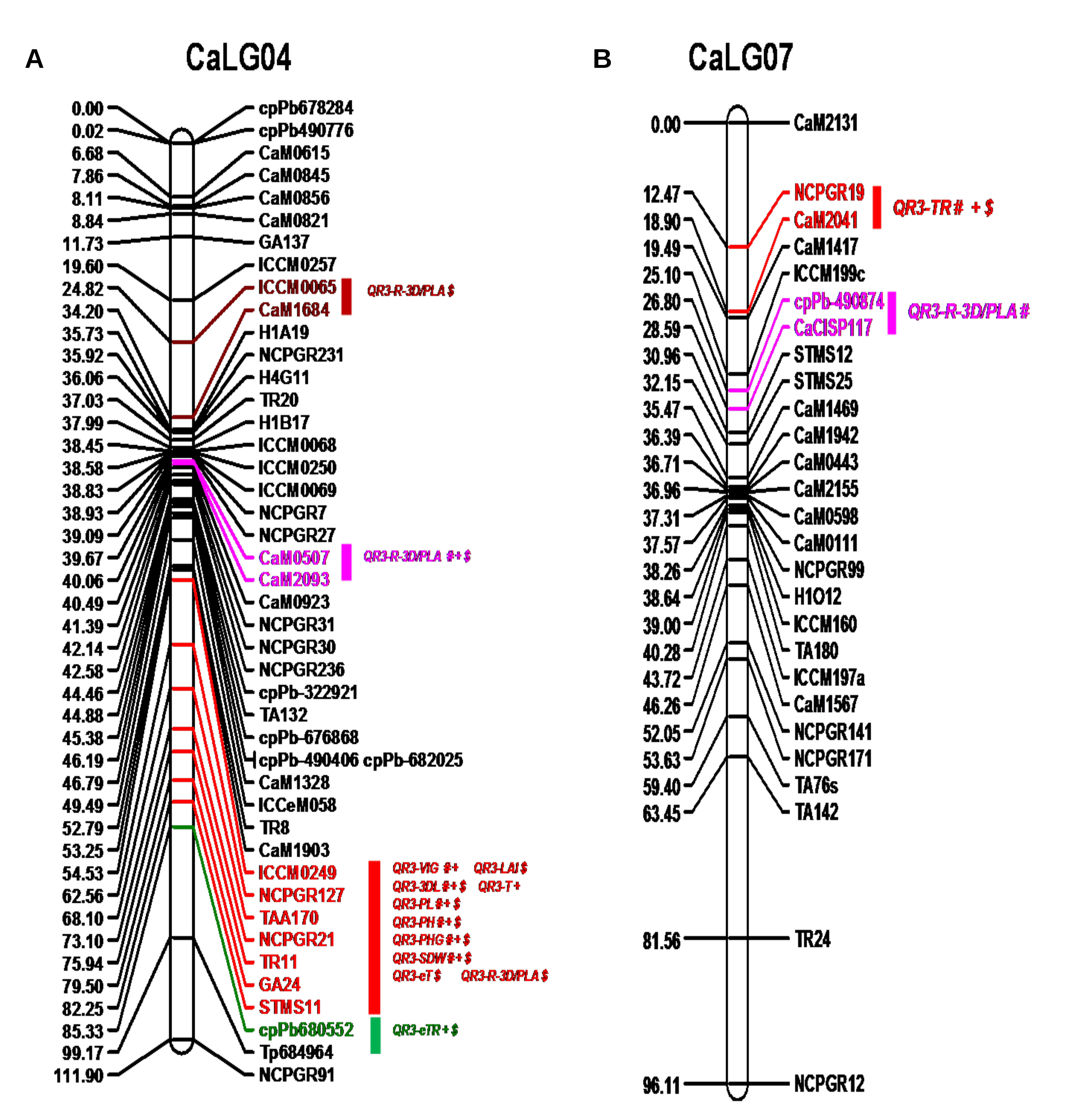

A
B

Supplement: Supplementary file 12 — Genetic map of chickpea RIL population derived from ICC 4958 x ICC 1882. The genetic map represents marker position and corresponding marker name in linkage group. Genetic distances (cM) were shown on the left and markers are shown on the right side of the bars. The map was constructed using Map chart software. The Q represents QTL and R3 represents the population name. Year of mapping was represented by the symbols: # (2014), + (2015) and $ (across the year). A represent CaLG04 with identified QTLs position and its corresponding marker. The markers of the QTLs regions within the hotspot (most plant vigour related traits) were represented in red and outside the hotspot were represented in pink (QR3R-3D/PLA # + $), green (QR3-eTR+ $) and brown (QR3R-3D/PLA $). B) Map represents CaLG07 with QTLs for transpiration rate (QR3-TR) (markers highlighted in red colour) and residuals from 3D and projected leaf area (QR3R-3D/PLA) (markers highlighted in pink colour). C) Map represents CaLG06 with QTLs for residuals from 3D and projected leaf area (QR3R-3D/PLA) (markers highlighted in red colour). D) Map represents CaLG08 with QTLs for transpiration (QR3-T) (markers highlighted in red colour). (ZIP 283 kb) [file 12870_2018_1245_MOESM12_ESM.zip › Additional file 12 A & B.pptx]

## Slide 1
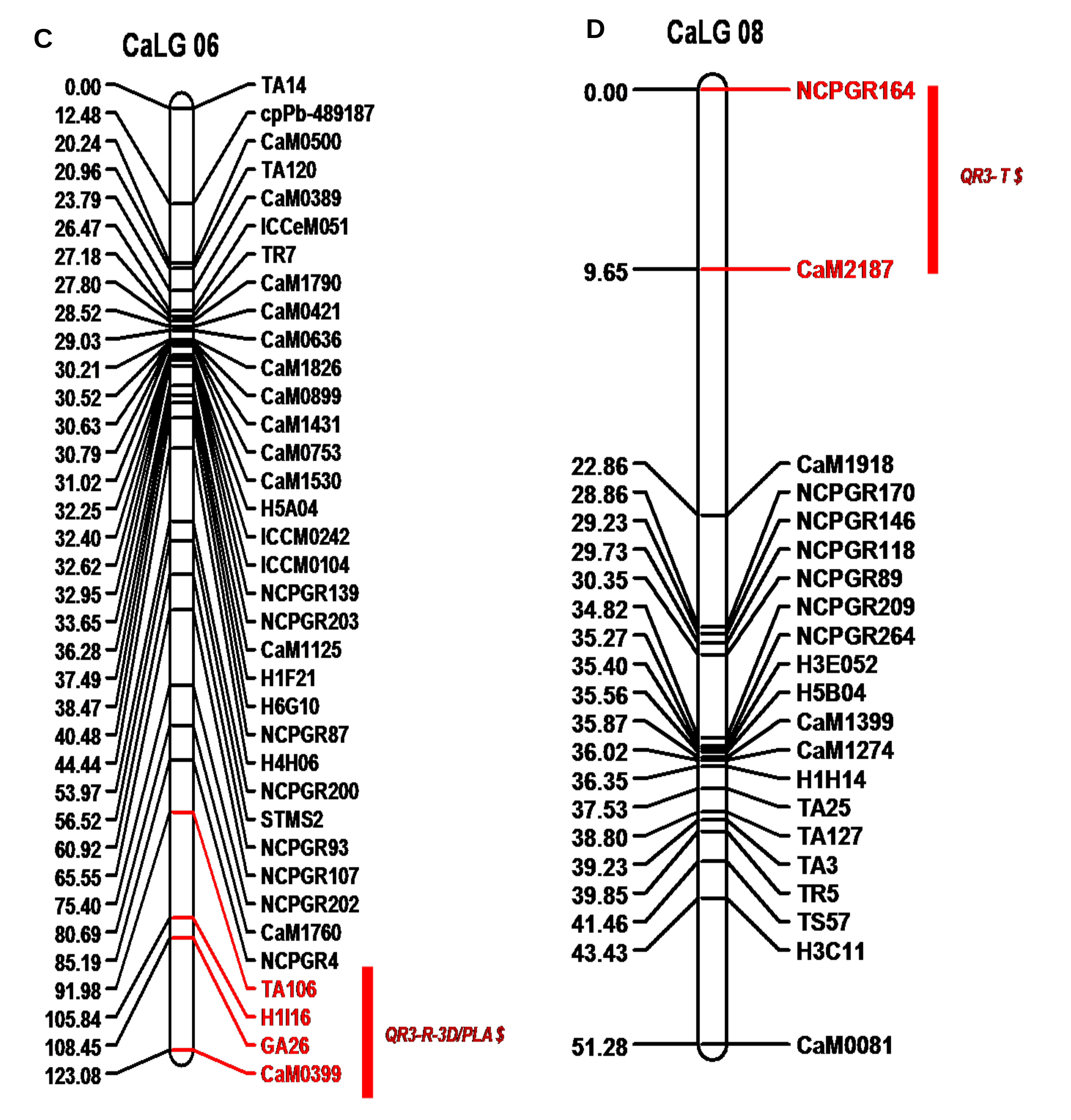

D
C

Supplement: Supplementary file 12 — Genetic map of chickpea RIL population derived from ICC 4958 x ICC 1882. The genetic map represents marker position and corresponding marker name in linkage group. Genetic distances (cM) were shown on the left and markers are shown on the right side of the bars. The map was constructed using Map chart software. The Q represents QTL and R3 represents the population name. Year of mapping was represented by the symbols: # (2014), + (2015) and $ (across the year). A represent CaLG04 with identified QTLs position and its corresponding marker. The markers of the QTLs regions within the hotspot (most plant vigour related traits) were represented in red and outside the hotspot were represented in pink (QR3R-3D/PLA # + $), green (QR3-eTR+ $) and brown (QR3R-3D/PLA $). B) Map represents CaLG07 with QTLs for transpiration rate (QR3-TR) (markers highlighted in red colour) and residuals from 3D and projected leaf area (QR3R-3D/PLA) (markers highlighted in pink colour). C) Map represents CaLG06 with QTLs for residuals from 3D and projected leaf area (QR3R-3D/PLA) (markers highlighted in red colour). D) Map represents CaLG08 with QTLs for transpiration (QR3-T) (markers highlighted in red colour). (ZIP 283 kb) [file 12870_2018_1245_MOESM12_ESM.zip › Additional file 12 C & D.pptx]
