# Supplementary material for: Plant vigour QTLs co-map with an earlier reported QTL hotspot for drought tolerance while water saving QTLs map in other regions of the chickpea genome
Source: BMC Plant Biol. 2018 Feb 6;18:29. doi: 10.1186/s12870-018-1245-1 (PMC5801699; doi:10.1186/s12870-018-1245-1)
Supplement: Supplementary file 2 — Frequency distribution of plant vigour and canopy conductance related traits. Frequency distribution of plant vigour (A, B, C & D) and canopy conductance (E, F, G & H) related traits in chickpea mapping population (ICC 4958 x ICC 1882) showing normal distribution. A, B, C & D represent the plant vigour, plant height, 3D-leaf area and shoot dry weight (Plant vigour related traits) and E, F, G & H represent transpiration rate, evapotranspiration rate, transpiration and evapotranspiration (Canopy conductance related traits). P1 and P2 represent the ICC 4958 and ICC 1882). (PPTX 220 kb) [file 12870_2018_1245_MOESM2_ESM.pptx]

## Slide 1
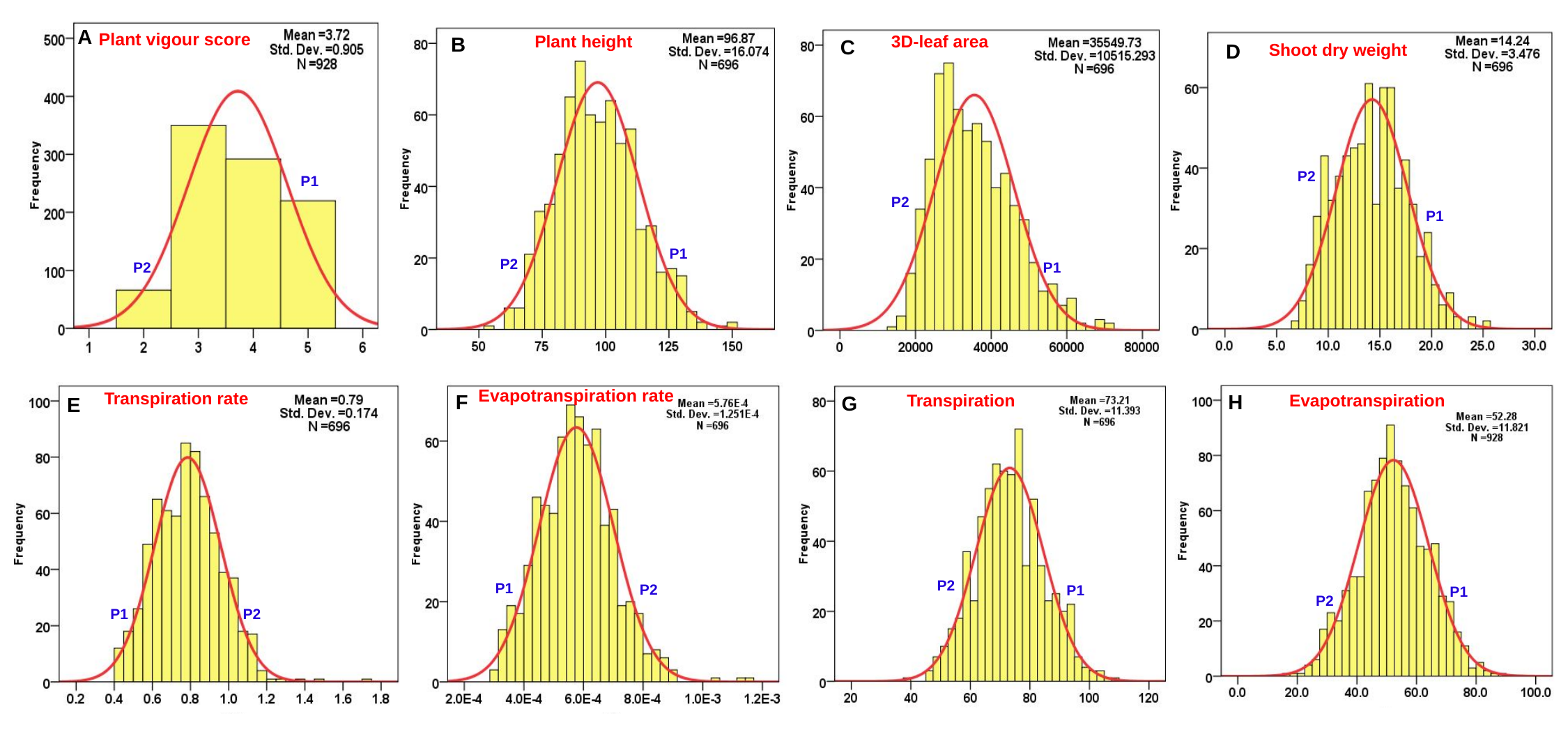

A
| | | | |
| --- | --- | --- | --- |
| | | | |
Plant vigour score
3D-leaf area
Plant height
B
C
D
Shoot dry weight
P2
P1
P2
P1
P1
P2
P2
P1
Evapotranspiration rate
Transpiration rate
F
Transpiration
H
Evapotranspiration
G
E
P2
P1
P2
P1
P1
P2
P2
P1
