# Supplementary material for: Plant vigour QTLs co-map with an earlier reported QTL hotspot for drought tolerance while water saving QTLs map in other regions of the chickpea genome
Source: BMC Plant Biol. 2018 Feb 6;18:29. doi: 10.1186/s12870-018-1245-1 (PMC5801699; doi:10.1186/s12870-018-1245-1)
Supplement: Supplementary file 3 — Growth dynamics of canopy development in contrasting parental lines. Growth dynamics of A) 3-leaf area and B) plant height in contrasting parental lines [High vigour parent (ICC 4958) and low vigour parent (ICC 1882) at vegetative stage calculated on the basis of thermal time (228-806 degree days for A and 114-806 degree days for B). (PPTX 181 kb) [file 12870_2018_1245_MOESM3_ESM.pptx]

## Slide 1
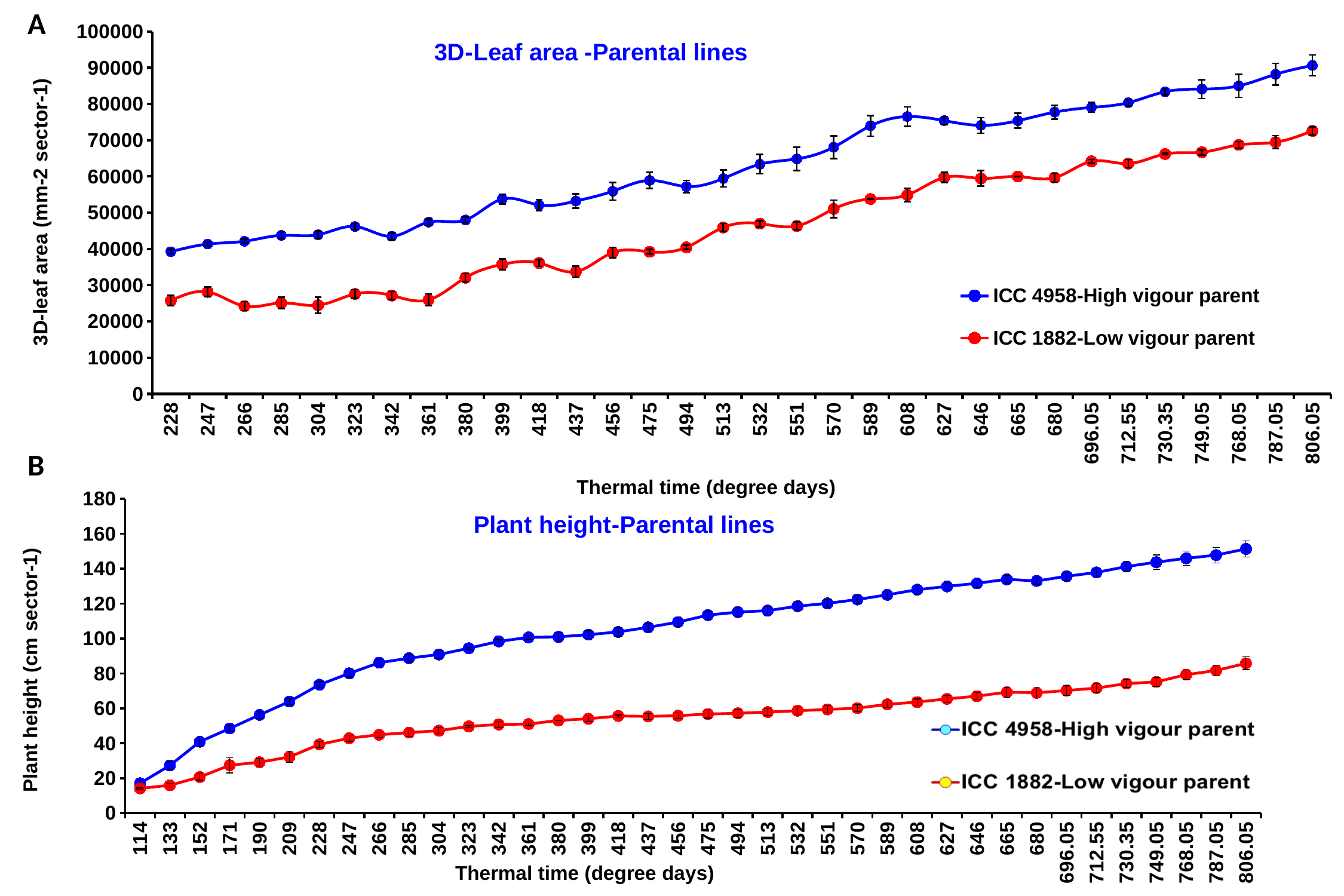

A
### Chart: 3D-Leaf area -Parental lines
| Category | | |
|---|---|---|
| 228 | 39243.33238095238 | 25751.311111111114 |
| 247 | 41335.710476190485 | 28150.12222222222 |
| 266 | 42120.433333333334 | 24213.544444444444 |
| 285 | 43745.509047619045 | 25101.255555555552 |
| 304 | 43920.89619047619 | 24470.777777777777 |
| 323 | 46188.83904761905 | 27576.655555555553 |
| 342 | 43487.14238095238 | 27145.78888888889 |
| 361 | 47397.36523809523 | 25953.7 |
| 380 | 47989.203095238096 | 32073.533333333333 |
| 399 | 53760.62238095239 | 35734.03333333333 |
| 418 | 52116.49 | 36119.211111111115 |
| 437 | 53218.71 | 33738.75555555555 |
| 456 | 55933.51142857143 | 39007.36666666667 |
| 475 | 58903.65285714285 | 39220.666666666664 |
| 494 | 57218.87666666667 | 40437.72222222222 |
| 513 | 59422.06619047619 | 45936.07777777778 |
| 532 | 63375.49380952381 | 46982.97777777778 |
| 551 | 64829.93476190476 | 46347.82222222222 |
| 570 | 68078.68476190475 | 51035.57777777778 |
| 589 | 73972.20857142858 | 53766.35555555557 |
| 608 | 76528.00333333334 | 54902.2 |
| 627 | 75414.88571428573 | 59706.677777777775 |
| 646 | 74107.42333333334 | 59456.62222222222 |
| 665 | 75390.08333333333 | 59969.76666666666 |
| 680 | 77745.43333333333 | 59658.94444444443 |
| 696.05 | 79054.2611904762 | 64162.72222222224 |
| 712.55 | 80363.08904761904 | 63547.59999999999 |
| 730.34999999999991 | 83380.59325396824 | 66190.7 |
| 749.05 | 84120.4661904762 | 66687.3111111111 |
| 768.05 | 85008.7642857143 | 68715.51111111112 |
| 787.05 | 88201.25619047618 | 69455.82222222222 |
| 806.05 | 90635.10571428573 | 72576.66666666667 || | |
| --- | --- |
| | |
B
### Chart: Plant height-Parental lines
| Category | ICC 4958-High Vigor parent | ICC 1882-Low Vigor parent |
|---|---|---|
| 114 | 17.04 | 14.120000000000001 |
| 133 | 27.27 | 15.9225 |
| 152 | 40.87166666666666 | 20.6475 |
| 171 | 48.416666666666664 | 27.3675 |
| 190 | 56.23666666666666 | 29.1 |
| 209 | 63.86166666666666 | 32.153333333333336 |
| 228 | 73.49166666666667 | 39.32 |
| 247 | 80.01833333333333 | 42.870000000000005 |
| 266 | 86.13833333333334 | 44.84333333333333 |
| 285 | 88.745 | 46.083333333333336 |
| 304 | 90.84000000000002 | 47.196666666666665 |
| 323 | 94.43166666666667 | 49.666666666666664 |
| 342 | 98.35333333333334 | 50.73 |
| 361 | 100.64166666666667 | 51.0 |
| 380 | 100.95916666666666 | 53.05166666666666 |
| 399 | 102.19333333333333 | 53.99666666666666 |
| 418 | 103.77833333333335 | 55.599999999999994 |
| 437 | 106.38916666666665 | 55.35 |
| 456 | 109.40499999999999 | 55.78333333333333 |
| 475 | 113.39166666666665 | 56.70666666666666 |
| 494 | 115.13833333333332 | 57.150000000000006 |
| 513 | 115.955 | 57.833333333333336 |
| 532 | 118.52166666666666 | 58.593333333333334 |
| 551 | 120.12166666666667 | 59.35666666666667 |
| 570 | 122.34333333333335 | 60.07000000000001 |
| 589 | 125.03500000000001 | 62.266666666666666 |
| 608 | 127.96499999999999 | 63.55333333333334 |
| 627 | 129.86333333333334 | 65.41333333333333 |
| 646 | 131.61333333333332 | 66.96333333333334 |
| 665 | 133.91166666666666 | 69.25999999999999 |
| 680 | 132.95833333333334 | 68.89 |
| 696.05 | 135.66 | 70.18 |
| 712.55 | 137.845 | 71.58999999999999 |
| 730.34999999999991 | 141.20166666666665 | 74.14666666666668 |
| 749.05 | 143.71333333333334 | 75.11 |
| 768.05 | 145.95 | 79.28 |
| 787.05 | 147.76833333333335 | 81.66333333333334 |
| 806.05 | 151.325 | 85.79333333333334 |
